# The Contribution of Optical Coherence Tomography in Neuromyelitis Optica Spectrum Disorders

**DOI:** 10.3389/fneur.2017.00493

**Published:** 2017-09-29

**Authors:** Javier Mateo, Olivia Esteban, Mireya Martínez, Andrzej Grzybowski, Francisco Javier Ascaso

**Affiliations:** ^1^Department of Ophthalmology, Hospital Clínico Universitario Lozano Blesa, Zaragoza, Spain; ^2^Department of Ophthalmology, Poznan City Hospital, Poznan, Poland; ^3^Chair of Ophthalmology, University of Warmia and Mazury, Olsztyn, Poland; ^4^Aragón Health Research Institute (IIS Aragón), Zaragoza, Spain

**Keywords:** neuromyelitis optica, optical coherence tomography, optic neuritis, multiple sclerosis, autoimmune diseases, Devic disease

## Abstract

Neuromyelitis optica spectrum disorders (NMOSD) comprises a group of central nervous system disorders of inflammatory autoimmune origin that mainly affect the optic nerves and the spinal cord and can cause severe visual and general disability. The clinical signs are similar to those of multiple sclerosis (MS), with the result that it is often difficult to differentiate between the two, thus leading to misdiagnosis. As the treatment and prognosis of NMOSD and MS are different, it is important to make an accurate and early diagnosis of NMOSD. Optical coherence tomography (OCT) is a non-invasive technique that enables a quantitative study of the changes that the optic nerve and the macula undergo in several neurodegenerative diseases. Many studies have shown that some of these changes, such as retinal nerve fiber layer thinning or microcystic macular edema, can be related to alterations in the brain due to neurodegenerative disorders. The purpose of this mini-review is to show how OCT can be useful for the diagnosis of NMOSD and follow-up of affected patients, as well as for the differential diagnosis with MS.

## Introduction

Neuromyelitis optica spectrum disorders (NMOSD), traditionally known as neuromyelitis optica or Devic disease, comprise a group of uncommon central nervous system (CNS) disorders of inflammatory autoimmune origin that mainly affect the optic nerves and/or the spinal cord (1–4).

Neuromyelitis optica spectrum disorders were first reported in the nineteenth century (5). Patients suffering from NMOSD can develop optic neuritis (ON) as well as transverse myelitis. ON, which is often the first manifestation of NMOSD, causes visual loss and painful eye movement, while myelitis can cause multiple alterations, such as sensory and movement disturbance, weakness, and loss of bowel and bladder control (1, 2, 5). Although these are the main clinical manifestations of the disease, other areas of the CNS can be affected, including the brain stem, diencephalon, *area postrema* on the dorsal surface of the medulla oblongata, and cerebrum (6–9). Alterations of the CNS due to NMOSD often generate severe visual and systemic disability (5, 10).

The clinical signs and symptoms of NMOSD are similar to those of multiple sclerosis (MS), thus making it difficult to perform a correct differential diagnosis (DD) between these two entities. As the treatment and prognosis of NMOSD and MS are different, it is important to make an accurate and early diagnosis of NMOSD (10). In general, ON is more severe and recurrent and more often bilateral in NMOSD than in MS. In addition, visual impairment is more severe, and recovery is poorer in NMOSD (10–13). The finding of aquaporin-4 immunoglobulin G (AQP4-IgG) in blood considerably facilitates diagnosis, as it is present in most patients with NMOSD, but not in MS (5, 10, 14, 15). Magnetic resonance imaging (MRI) findings, such as optic chiasm involvement in ON or alteration of at least three contiguous segments of the spinal cord in transverse myelitis, are highly suggestive of NMOSD (16–18). A newer nomenclature of NMOSD is now being used to include patients with clinical syndromes and/or MRI findings with or without AQP4-IgG. ON is not always present in NMOSD. In addition, antibodies against myelin oligodendrocyte glycoprotein (MOG-IgG) are found in one-third of patients with AQP4-IgG-seronegative NMOSD. MOG-IgG antibodies are useful for the DD with MS. Furthermore, disease course is usually more favorable in patients with AQP4-IgG-seronegative and MOG-IgG-seropositive NMOSD (19, 20).

Optical coherence tomography (OCT) is a non-invasive imaging technique that was created in the 1990s and is currently used worldwide. It enables an *in vivo* quantitative and qualitative study of changes in the optic nerve and the macula (10, 21). It is fast, comfortable for the patient, and easy to perform and has, therefore, been used for several years to study many ophthalmic diseases, mainly glaucoma and macular disorders. In neuro-ophthalmology, OCT is considered a “window” to the CNS, and is widely used to study neurodegenerative diseases such as MS, NMOSD, Alzheimer’s disease, and Parkinson’s disease (10, 22, 23).

In this mini-review, we show how OCT can lead to a faster and more accurate diagnosis and follow-up of NMOSD and also how it can help to differentiate NMOSD from other neurodegenerative disorders, especially MS.

## Methods

We performed a literature search in PubMed in April 2017 using the terms “neuromyelitis optica” and “neuromyelitis optica spectrum disorders” combined with “optical coherence tomography” or “OCT.” We looked for original case-control studies, case series, or cohort studies, as well as reviews on the topic “neurodegenerative disorders,” “multiple sclerosis,” “neuromyelitis optica,” “optic neuritis,” or “optic neuropathy.” We also reviewed many of the references of the articles found.

## Results

**Figure 1:**
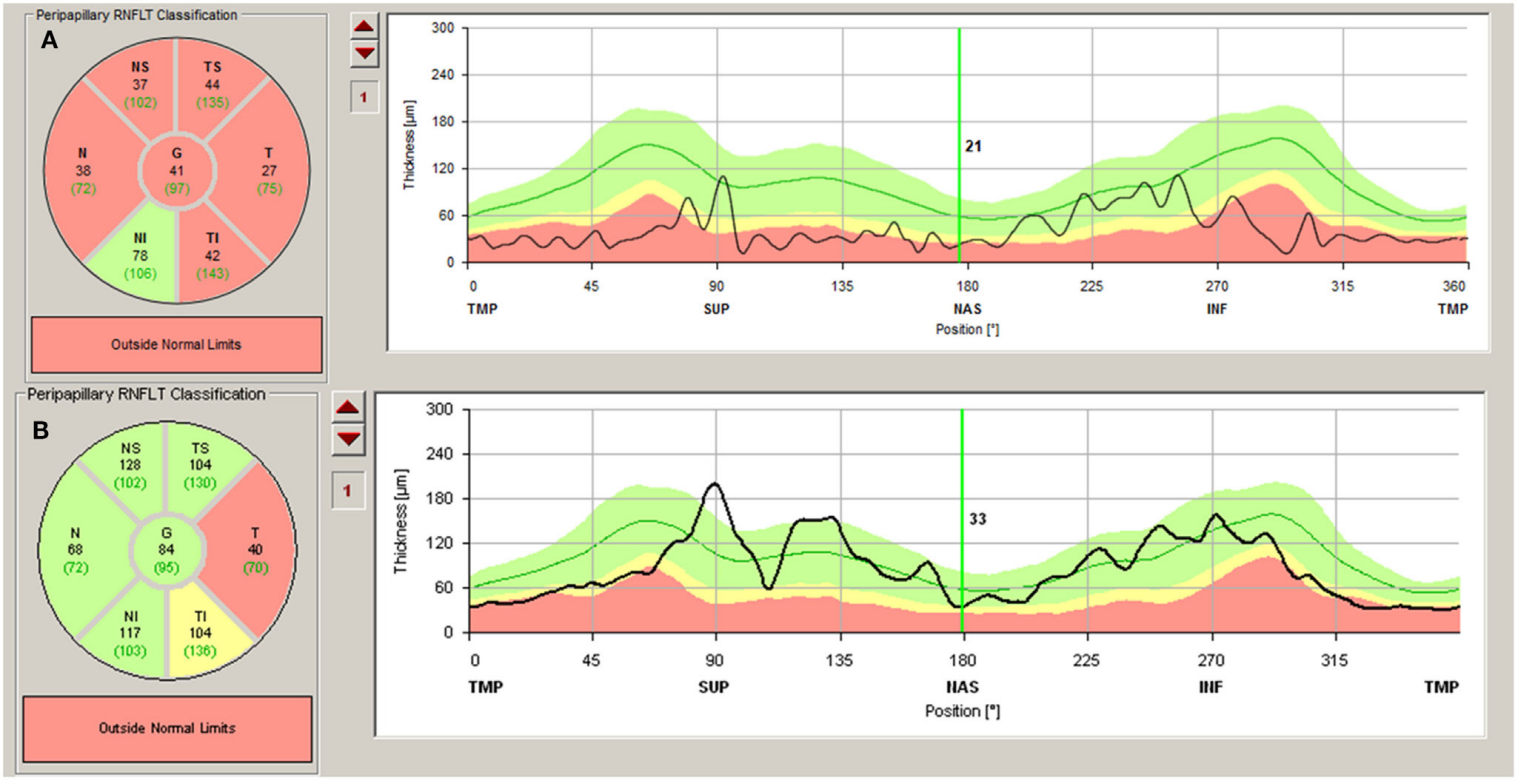
**(A)** Severe atrophy of peripapillary retinal nerve fiber layer (pRNFL) after optic neuritis (ON) in a neuromyelitis optica spectrum disorders patient. **(B)** Atrophy of pRNFL temporal quadrant in a multiple sclerosis patient without history of ON.

The most usual finding when OCT is used in patients with NMOSD is thinning of the peripapillary retinal nerve fiber layer (pRNFL). NMOSD studies, some cross-sectional studies, and cohort and prospective longitudinal studies show a marked decrease in pRNFL thickness after ON (11, 15, 23–27). As this finding is also observed in ON associated with other disorders, it does not facilitate the DD. However, many authors report that the decrease in pRNFL thickness and also in macular volume is more pronounced after ON in NMOSD than in MS (Table 1) (11, 15, 28–34). This could be explained by the fact that episodes of ON in NMOSD tend to be more severe and recovery poorer than in MS (Figure 1A) (11, 29, 30).

**Table 1:** Average peripapillary retinal nerve fiber thickness (in micrometers) after optic neuritis in patients with neuromyelitis optica spectrum disorders (NMOSD), multiple sclerosis (MS), and healthy controls.

		NMOSD	MS	Controls
Ratchford et al. (28)	Range	31–99.4	44.4–129.5	80.8–128.1
	Mean ± SD	63.6 ± 20.3	88.3 ± 16.5	102.4 ± 11.0
Schneider et al. (29)	Range	28–93.4	62.3–105.2	80.7–122.3
	Mean ± SD	58.5 ± 21.2	85.3 ± 13.3	100.1 ± 10.8
Manogaran et al. (30)	Mean ± SD	70.8 ± 16.4	81.0 ± 12.8	100.1 ± 10.8
Tian et al. (33)	Mean ± SD	63.94 ± 11.86	79.12 ± 15.64	112.01 ± 10.93
Naismith et al. (35)	Mean ± SD	54.8 ± 3.7	76.5 ± 2.4	
Park et al. (36)	Mean ± SD	49.53 ± 3.81	70.09 ± 5.78	100.08 ± 9.34

When differences in thinning of pRNFL are compared between NMOSD and MS, the damage in NMOSD affects every quadrant, mainly the superior and inferior quadrant, while in MS, it is more severe in the temporal quadrant (15, 24, 32, 35). Some authors state that this alteration of the different sectors of the pRNFL is caused by damage to the small-diameter axons—located mainly in the temporal quadrant—in MS and a more diffuse injury in NMOSD (15, 24).

Another frequent alteration highlighted by OCT in NMOSD is the reduced thickness of the ganglion cell layer (GCL) in the macula (10, 11, 15, 33, 36). This alteration has also been described in other neurodegenerative diseases, such as Alzheimer’s disease and MS (10, 37). However, it seems that the decrease in the thickness of the GCL is more pronounced in NMOSD owing to intense inflammation and necrosis, with more prominent neuronal and axonal damage in NMOSD than in MS (11, 36). The more severe thinning of the superior quadrant of the GCL, which is similar to that observed in anterior ischemic optic neuropathy (AION), suggests vascular and ischemic damage to the optic nerve in NMOSD (36, 38). Recent investigations show foveal thinning in NMOSD patients with or without ON, indicating damage to the retina, perhaps because Müller cells and retinal astrocytes expressing AQP4 are targeted in NMOSD, even when there is no history of ON (39–41).

In some cases of NMOSD, microcystic macular edema can affect the inner nuclear layer of the retina after ON, a feature that has also been described in other conditions of the optic nerve, such as Leber hereditary optic neuropathy, AION, MS, compressive or traumatic optic neuropathy, hydrocephalus, and even glaucoma. The origin of this edema remains unclear, although the main hypotheses are trans-synaptic degeneration and vitreous traction (15, 21, 24, 42, 43).

When ON is not present in NMOSD, there also seem to be differences between MS and NMOSD. While in MS there is significant subclinical thinning of the pRNFL, especially in the temporal quadrant, this damage does not occur or is very subtle in NMOSD without ON (Figure 1B) (15, 26–29). Nevertheless, there does seem to be a subclinical decrease in the thickness of the GCL and macular thickness in NMOSD without ON (11, 15, 24, 30, 44, 45). This might be caused by damage of the Müller cells in the retina (see above). These cells are rich in AQP4 channels, which are attacked in NMOSD (11, 45).

A relationship can be observed between OCT changes and clinical features in NMOSD. For example, the decrease in pRNFL thickness and macular volume is apparently associated with the degree of visual disability in NMOSD, and the same can be observed with the decrease in the thickness of the GCL. Similar effects can be observed in MS (10, 15, 24, 33, 46). Although while in MS there is a clear correlation between the reduction in pRNFL and GCL thickness and general disability, this correlation has not been reported as frequently in NMOSD (15, 26, 47–50).

## Discussion

Although OCT alone cannot confirm the DD between NMOSD and MS, it is highly accurate and plays a useful role in the follow-up of NMOSD.

The diagnostic criteria for NMOSD include clinical features (mainly ON and acute myelitis), the presence of AQP4-IgG in most patients, and characteristic MRI findings (3).

Owing to the similarities between NMOSD and MS, it is sometimes difficult to discriminate between both entities. Combination of OCT and the aforementioned diagnostic criteria can enable a more precise DD.

During the acute and early phase of ON, when the optic nerve head is swollen and the pRNFL does not provide reliable data, OCT is particularly useful for assessment of the GCL, which is frequently more severely affected in NMOSD than in MS (33).

Other OCT changes that can facilitate the DD are thinning of the pRNFL and GCL and reduction in macular volume, which are usually more severe in NMOSD than in MS after ON (11, 15). These changes, which predominantly affect the superior and inferior quadrants, are suggestive of NMOSD, while damage in the temporal quadrant points to MS (15, 24).

In cases of suspicion of NMOSD without ON, the thinning of the temporal quadrant of the pRNFL is characteristic of MS rather than NMOSD, since in the latter, pRNFL is only slightly affected or unaffected (15, 24).

Because of the relationship between damage to visual function and the decrease in pRNFL, macular volume, and the GCL, OCT could prove to be a valuable instrument for the follow-up of NMOSD (10, 15, 24).

Further studies are necessary to provide more evidence and show us the extent of the usefulness of OCT for the diagnosis, study, and follow-up of NMOSD.

## Author Contributions

JM, OE, and MM: concept, design, collection of data, writing MS, and discussion. AG and FA: concept, design, and discussion.

## Conflict of Interest Statement

The authors declare that the research was conducted in the absence of any commercial or financial relationships that could be construed as a potential conflict of interest.
